# Chances and Limits of the Coordination Chemistry with Bis(benzene-l,2-dithiolato) Ligands

**DOI:** 10.1155/BCA.2005.69

**Published:** 2005

**Authors:** Wolfram W. Seidel, F. Ekkehardt Hahn

**Affiliations:** Institut für Anorganische und Analytische Chemie Westfälische Wilhelms-Universität Münster Wilhelm-Klemm-Straße 8 Münster D-48149 Germany

## Abstract

The incorporation of benzene-l,2-dithiolato building blocks into supramolecular coordination assemblies is the main objective of the investigations described here. Special interest is directed towards dinuclear complexes with bis(benzene-l,2-dithiolato) ligands, which might be able to form helical structures. Bis(benzene-l,2-dithiolato) ligands are accessible by *ortho*-functionalization and subsequent linkage of two benzene-l,2-dithiol units. The preparation of well defined complexes of titanium, cobalt and nickel with bis(benzene-l,2-dithiolato) ligands requires strictly thermodynamic equilibration conditions. In that case the size and shape of the ligand backbone determine if dinuclear double-stranded or mononuclear chelate complexes are obtained. The dinuclear double-stranded complexes with Ni(II) and Ni(III) are characterized by a coplanar non-helical arrangement of the square-planar bis(benzene-l,2-dithiolato)nickelate moieties. The complete structural characterization of the series [M(C_6_H_4_S_2_ - 1,2)_3_]^n^ - (n = 0, 1, 2) for molybdenum and tungsten indicates an interesting coordination chemistry of dinuclear triple-stranded complexes.


### Chances and Limits of the Coordination Chemistry with Bis(benzene-1,2-dithiolato) Ligands

Wolfram W. Seidel, F. Ekkehardt Hahn*

*Institut für Anorganische und Analytische Chemie, Westfälische Wilhelms-Universität Münster*

*Wilhelm-Klemm-Straße 8, D-48149 Münster (Germany)*

*FAX: (+49)-251-8333108*

*e-mail: fehahn@uni-muenster.de*

### ABSTRACT

The incorporation of benzene-1,2-dithiolato building blocks into supramolecular coordination assemblies is the main objective of the investigations described here. Special interest is directed towards dinuclear complexes with bis(benzene-1,2-dithiolato) ligands, which might be able to form helical structures. Bis(benzene-1,2-dithiolato) ligands are accessible by *ortho*-functionalization and subsequent linkage of two benzene-1,2-dithiol units. The preparation of well defined complexes of titanium, cobalt and nickel with bis(benzene-1,2-dithiolato) ligands requires strictly thermodynamic equilibration conditions. In that case the size and shape of the ligand backbone determine if dinuclear double-stranded or mononuclear chelate complexes are obtained. The dinuclear double-stranded complexes with Ni(II) and Ni(III) are characterized by a coplanar non-helical arrangement of the square-planar bis(benzene-1,2-dithiolato)nickelate moieties. The complete structural characterization of the series $[M(C_6H_4S_2\text{-}1,2)_3]^{n-}$ (n = 0, 1, 2) for molybdenum and tungsten indicates an interesting coordination chemistry of dinuclear triple-stranded complexes.

### INTRODUCTION

The coordination chemistry of unsaturated 1,2-dithiolato ligands has been studied thoroughly during the past decades /1/. The remarkable electronic properties uncovered and the structural features of benzene-1,2-dithiolato complexes /2/ prompted us to launch a systematic investigation aimed to incorporate benzene-1,2-dithiolato building blocks into supramolecular coordination assemblies. Our approach is based on the *ortho*-functionalization of benzenedithiol and the subsequent linkage of benzene-1,2-dithiol units by suitable spacers either to itself or alternatively to other ligand moieties with different donor centers. On the one hand we hope, sterically restricted macrocyclic ligands are able to stabilizes long sought after, low molecular weight analogues of polynuclear, sulfur-rich active centers of metal containing enzymes. Our particular interest in this field is directed towards dinuclear iron sites containing $Fe_2S_2$ units like mixed valent [2Fe-2S]-ferredoxins /3/ and "Rieske" centers /4/. On the other hand we are interested in general aspects of metal based supramolecular chemistry with poly(dithiolato) ligands. In this context the ability of polydentate ligands to form helical coordination compounds by self-assembly with metal ions is inspiring. Whereas early helicate chemistry was dominated by oxygen /5/ and nitrogen /6/ containing ligands, to the best of our knowledge no helicates with benzene-1,2-dithiolato donor centers are known so far. Triple-stranded helicates with nitrogen or oxygen donors exhibit two chiral metal centers, which are normally coordinated in an octahedral fashion. In contrast to this, tris(benzene-1,2-dithiolato) complexes form either trigonal-prismatic or rather octahedral coordination polyhedra depending on the metal, its oxidation state and the reaction conditions. This unique behavior of benzene-1,2-dithiolato complexes points out the intriguing opportunity to develop dinuclear coordination compounds, in which the helicity might be switched on or off by either electron transfer or the application of chiral conditions, that causes a change in the coordination geometry from achiral $D_{3h}$ to chiral $O_h$. Starting in 1995 we reported on the synthesis and coordination chemistry of various amide- and alkyl-bridged tris- and bis(benzene-1,2-dithiolato) ligands /7/. Herein we focus on our recent efforts to synthesize dinuclear complexes stabilized by bis(benzene-1,2-dithiolato) ligands (Figure 1).

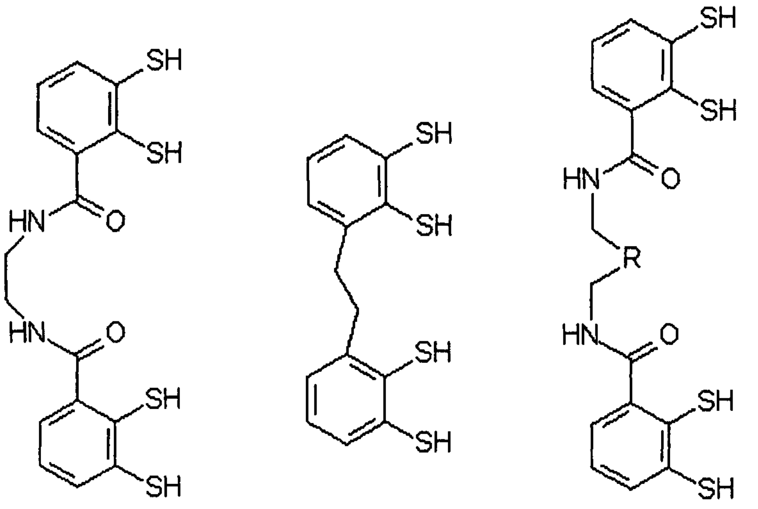

$H_4$-**6** $H_4$-**7** R = 1,4-$C_6H_4$ $H_4$-**8**
R = $(CH_2)_5$ $H_4$-**9**
R = $CH_2$ $H_4$-**10**

**Fig. 1:** Survey of the ligands discussed herein.

### LIGAND SYNTHESIS

The bridging of benzene-1,2-dithiol building blocks by a desirably broad variety of highly stable backbones can be achieved either *via* amide or even better by alkyl linkage. The key step in this approach to bis(benzene-1,2-dithiol) derivatives is the suitable functionalization of benzene-1,2-dithiol by *ortho*-lithiation and subsequent reaction either with carbon dioxide leading to 2,3-dimercaptobenzoic acid or with paraformaldehyde resulting in the formation of the corresponding benzyl alcohol (Scheme 1). In our search for an efficient synthetic procedure, we found lithium 2,3-bis(isopropylmercapto)benzene **1** (Scheme 1) to be an excellent precursor /7d, 7e/. Compound **1** can be prepared by *ortho*-lithiation of 1,2-di(isopropylmercapto)benzene, which is most conveniently obtained from *o*-dichlorobenzene and sodium isopropylmercaptane in DMF, with one equivalent *n*-butyllithium in the presence of TMEDA. Reaction of **1** with dry $CO_2$ followed by hydrolysis gave nearly quantitatively the desired product 2,3-di(isopropylmercapto) benzoic acid **2**. However, reaction of the lithiated species **1** with paraformaldehyde provided the benzyl alcohol, which can be converted into the bromide **3** by treatment with $PBr_3$. The bridging of two or three moleculs of **2** with different di- or triamides by standard methods gave very stable bis- or trisamides in analogy to 1,2-bis[2,3-di(isopropylmercapto)benzamido]ethane **4** shown in scheme 1. The Wurtz-type coupling reaction of bromide **3** however lead to the solely carbon bridged species **5**. In all cases the free ligands can be obtained by cleavage of the S-alkyl bonds with sodium/naphthalene in THF and subsequent hydrolysis as off-white powders. A selection of ligands prepared by the routes described here are shown in Figure 1.

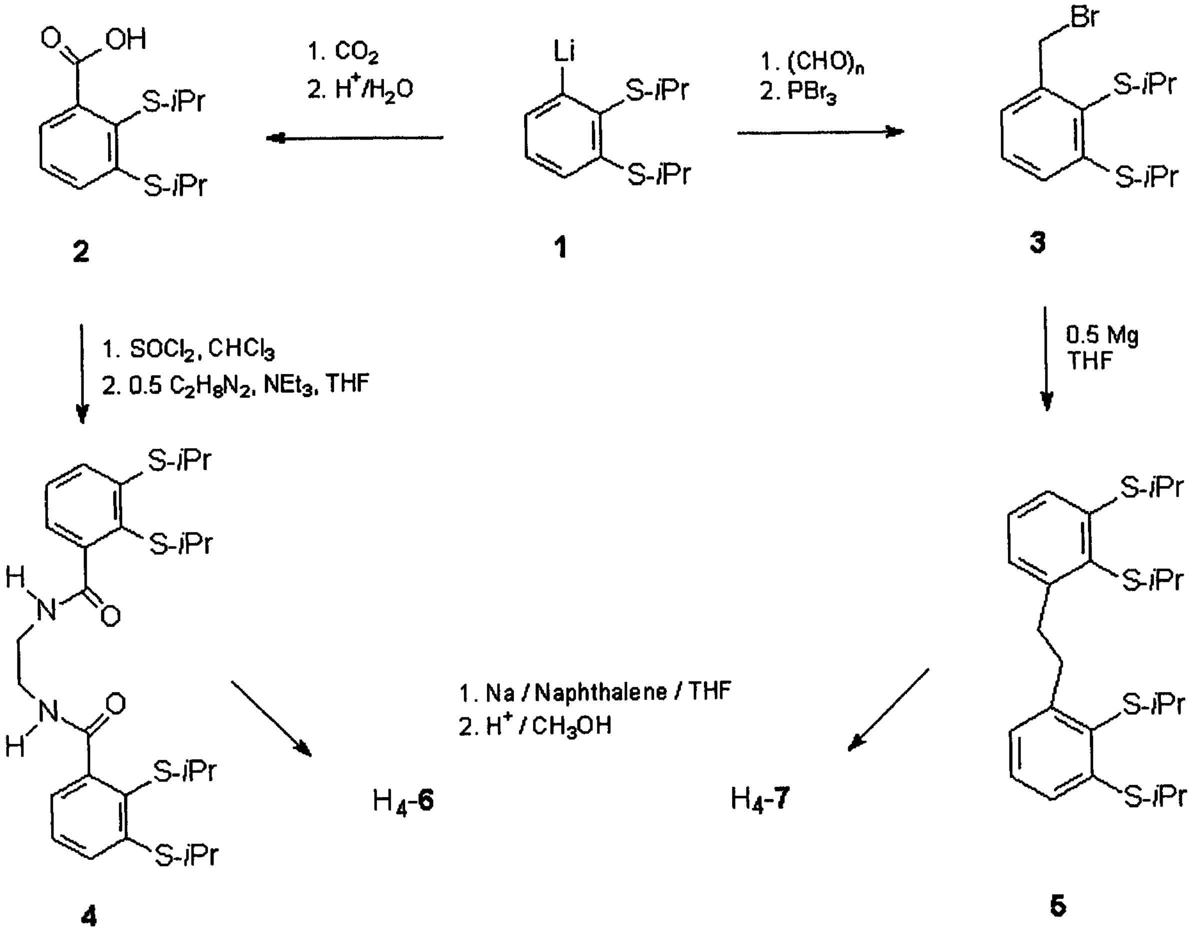

**Scheme 1:** Synthesis of the ligands $H_4$-**6** and $H_4$-**7**.

In contrast to simple, unbridged 2,3-dimercaptobenzamides, the bis(benzene-1,2-dithiols) are only sparingly soluble in common organic solvents like tetrahydrofuran, dichloromethane, acetonitril, acetone or methanol. Whereas the ligands can only be dissolved in dimethylformamide or dimethylacetamide the solubility in protic solvents like water and alcohols is very much improved by deprotonation. Obviously the intermolecular hydrogen bonding is crucial for the poor solubility; however, this is due not to the strength but to the high number of such interactions. The poor solubility of bis(benzene-1,2-dithiol) derivatives turned out to be critical for the study of their coordination chemistry, because the highly charged bis(benzene-1,2-dithiolato) ligands react very fast and act as strong reducing agents. To overcome this limitation we designed and synthesized ligand $H_4$-**7** (Figure 1) /7d/, the first example of an alkyl bridged bis(benzene-1,2-dithiol) ligand. Ligand $H_4$-**7** is freely soluble in most common organic solvents such as tetrahydrofuran and dichloromethane. In the case of **5** the linkage of the two benzene-1,2-dithiol units has been proven by X-ray crystal structure analysis /7e/.

### CHELATE COMPLEXES

The size and shape of the bis(benzene-1,2-dithiolato) ligand backbone determines if dinuclear double stranded or mononuclear chelate complexes are obtained. In addition, the topology of all bis(benzene-1,2-dithiolato) ligands synthesized also allows the formation of oligonuclear species or even complex polymers. Therefore fast complex formation under kinetically controlled conditions is undesired. Usually, bis(benzene-1,2-dithiolato) complexes are prepared by simple metathesis reaction of highly reactive alkali dithiolates and metal halides by salt elimination. A slow and mild alternative working exclusively at elevated temperatures was found to be the ligand transfer reaction of titanocene benzene-1,2-dithiolato complexes with tetrachlorometalates. Particularly the formation of mononuclear complexes has been achieved by the convenient transfer reaction of titanocene complexes with a bis(benzene-1,2-dithiolato) ligand with $[NiCl_4]^{2-}$ or $[CoCl_4]^{2-}$ (Scheme 2). The bis(benzene-1,2-dithiolato) ligand $[\mathbf{8}]^{4-}$ with the short and rigid xylylen bridge is capable of forming the chelate complex $(NMe_4)[(\eta^5\text{-}C_5H_5)Ti(\mathbf{8})]$ **11** (Figure 2) /7b/. The cobalt(III) complex with $[\mathbf{8}]^{4-}$ turned out to be unstable with respect to its coordination polymer. A stable bis(benzene-1,2-dithiolato)cobaltate(III) complex **12** (Figure 2) has been obtained with the longer and more flexibly bridged ligand $[\mathbf{9}]^{4-}$ /7c/. The difference in the behaviour between $[Co(\mathbf{8})]^-$ and $[Co(\mathbf{9})]^-$ is reflected in the UV/Vis and cyclic voltammetry spectra /7c/. Consequently the electronic properties and the reactivity of the metal center in bis(benzene-1,2-dithiolato) complexes with bridged dithiolato units are influenced by the character of the bridge, thus by effects originating relatively far from the metal center in the organic backbone of the ligand. In addition, the sterically restricting character of $[\mathbf{8}]^{4-}$ is clearly visible in the molecular structure of **11**. The coordination sphere around the titanium in the complex anion $[(\eta^5\text{-}C_5H_5)Ti(\mathbf{8})]^-$ is distorted compared to that in $[(\eta^5\text{-}C_5H_5)Ti(S_2C_6H_4)_2]^-$ /8/. Owing to the steric requirements of the xylylen bridge the endo bend of one chelate ring is much larger than in the prototype complex and the interdithiolate separation is distinctly shorter on the side of the bridge (S1-S4 compared to S2-S3).

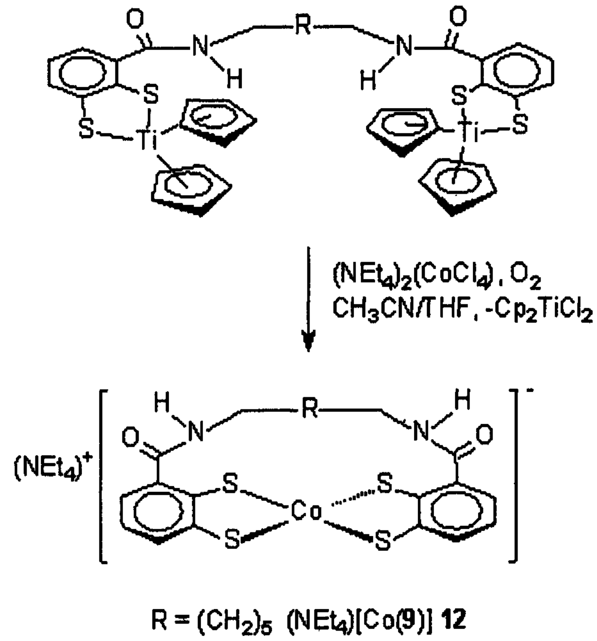

**Scheme 2:** Complex synthesis using the titanocen transfer reaction with $[CoCl_4]^{2-}$.

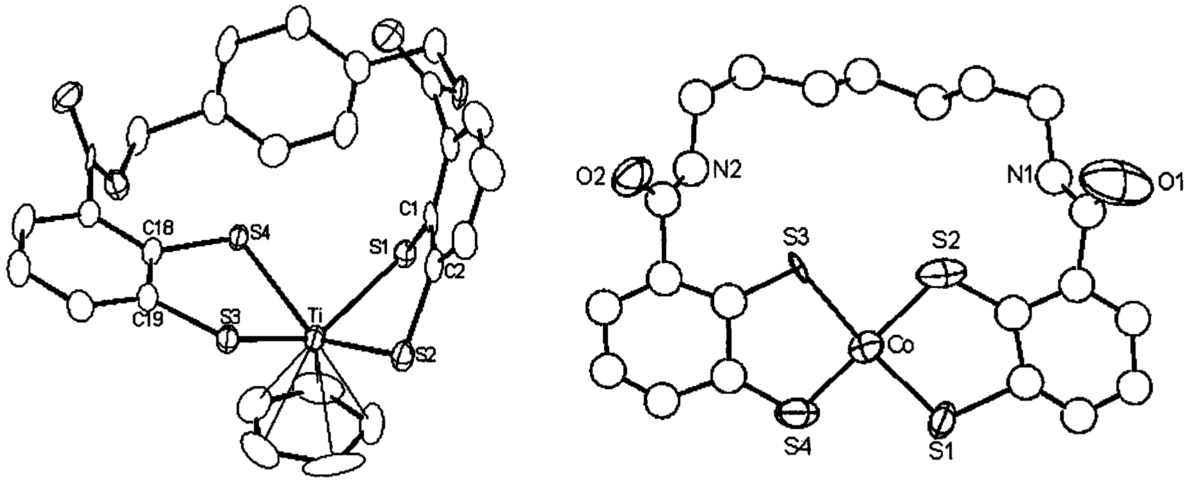

**Fig. 2:** Molecular structures of the complex anions in **11** (left) and **12** (right).

### DOUBLE STRANDED DINUCLEAR COMPLEXES

During efforts to optimize the reaction pathway to dinuclear double-stranded bis(benzene-1,2-dithiolato) nickelate complexes we found both routes, simple metathesis with sodium salts of the ligands and the titanocene transfer reaction, are suitable, when low temperature and short reaction times are avoided. Treatment of $NiCl_2$ with either $Li_4$-**6** or $Li_4$-**7** (Figure 1) in a 1:1 stoichiometry in methanol under reflux conditions and subsequent metathesis with $NEt_4Cl$ afforded air sensitive microcrystalline $(NEt_4)_4[Ni^{II}_2(\mathbf{6})_2]$ **13a** or $(NEt_4)_4[Ni^{II}_2(\mathbf{7})_2]$, **14a**, respectively /7d/. The same products can be prepared by ligand transfer reaction of the neutral dinuclear bis(titanocene) complexes with $(NEt_4)_2[NiCl_4]$ in an acetonitrile/ tetrahydrofuran solvent mixture under reflux conditions. Aerial oxidation of **13a**/**14a** as indicated by color change from dark brown to intense green led to $(NEt_4)_2[Ni^{III}_2(\mathbf{6})_2]$ **13b** or $(NEt_4)_2[Ni^{III}_2(\mathbf{7})_2]$ **14b**, respectively.

The structure analyses of **13a**, **13b** (Figure 3) and **14a** (Figure 4) revealed for all three complexes discrete dinuclear bis(benzene-1,2-dithiolato) nickelate complex anions /7d/. Each complex anion contains two square planar $[Ni(S_2C_6H_3R)_2]$ units with the nickel centers coordinated by two benzene-1,2-dithiolate units from different ligands in a square-planar fashion. Both $[Ni(S_2C_6H_3R)_2]$ moieties are linked in a double-stranded fashion by the backbones of the ligands. The amide N-H group in catechoylamide complexes are normally

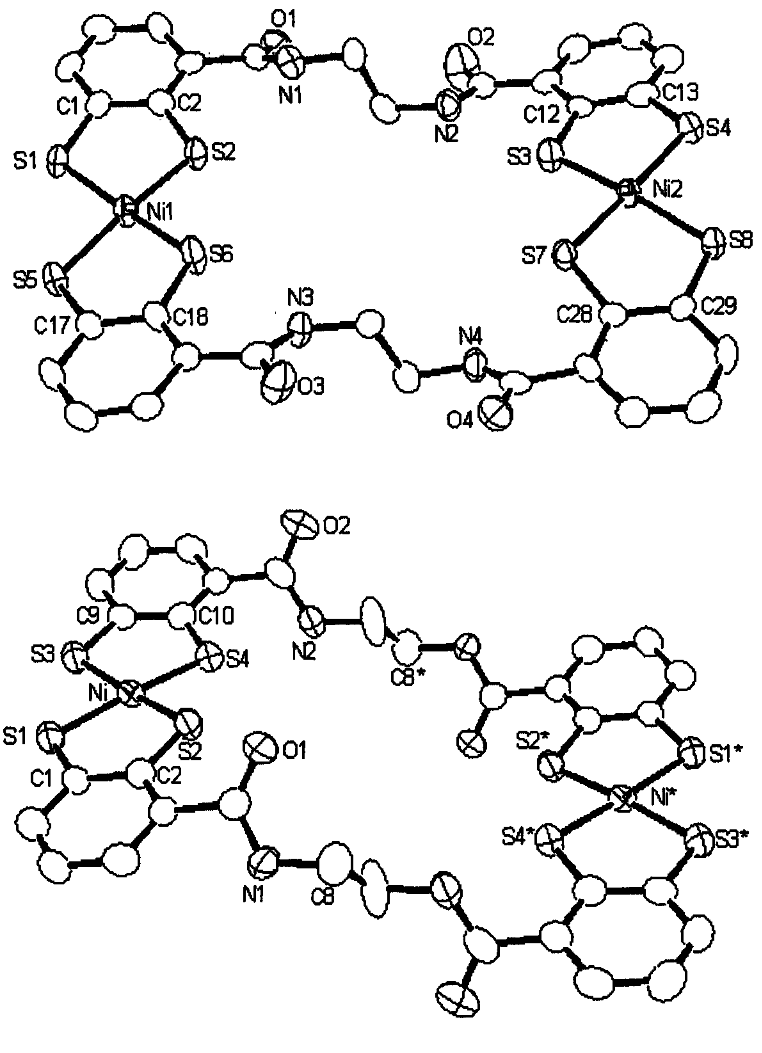

**Fig. 3:** Molecular structures of the nickelate anions in $(NEt_4)_4[Ni^{II}_2(\mathbf{6})_2]$ **13a** (top) and $(NEt_4)_2[Ni^{III}_2(\mathbf{6})_2]$ **13b** (bottom).

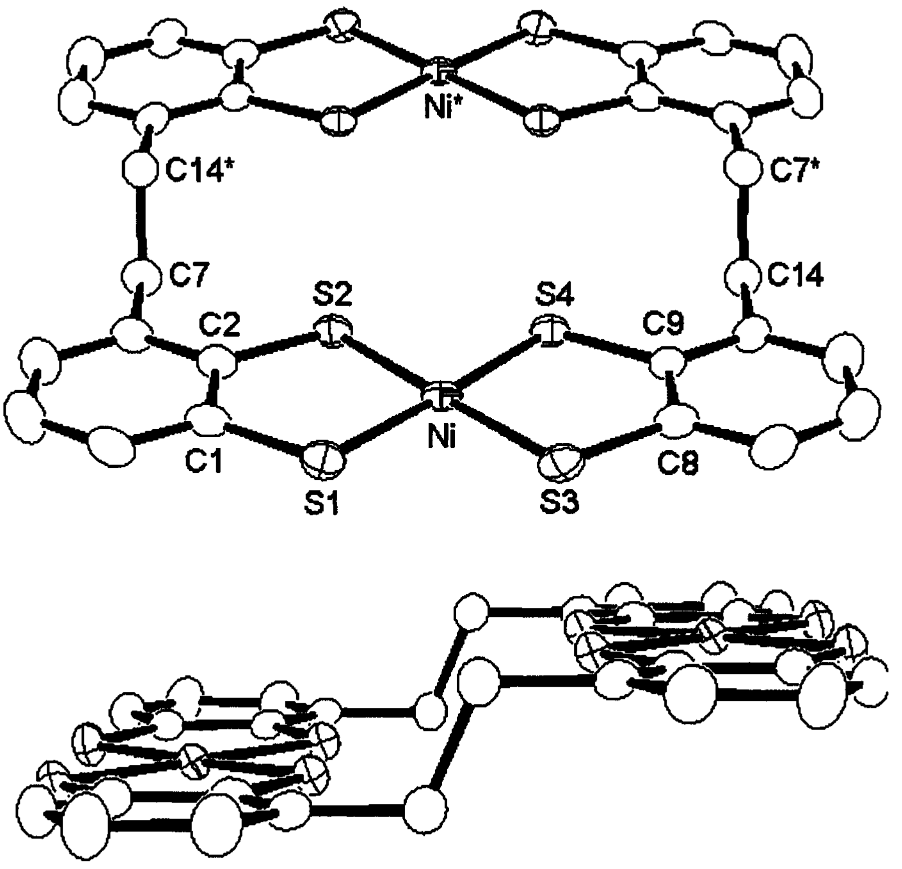

**Fig. 4:** Molecular structure of the nickelate anion in $(NEt_4)_4[Ni^{II}_2(7)_2]$, **14a** (top) and of its stair-type conformation (bottom).

involved in a hydrogen bond to the *o*-oxygen atom of the catechol /5/. In contrast to the dianion **13b**, a similar behaviour was observed in the tetraanion of **13a**, in which all amide N-H groups point to the *o*-sulfur atom of the benzene-1,2-dithiolate. We were particularly interested to find out how such secondary effects together with the sterical requirements of the amide functions of the spacer could lead to a double-stranded helical molecular structure. For such a helicity in a dinuclear complex it would be required that the $MS_4$ planes are arranged in a non-coplanar fashion and are twisted around the metal-metal vector. The structure analyses, however, showed for all three complexes a stair-like arrangement of the $NiS_4$ planes. This is illustrated in Figure 4 for complex **14a** with exactly coplanar $NiS_4$ moieties. The distance between the two $NiS_4$ units in the anions of **13a** and **13b** is longer than in **14a** owing to the longer ethylenediamide bridges. The nickel-nickel distance in **14a** amounts to 6.72 Å compared to 10.89 Å for **13a** and 10.96 Å for **13b**.

In cyclovoltammetric studies with the $[Ni_2(6)_2]^{2-/4-}$ and $[Ni_2(7)_2]^{2-/4-}$ couples quasi-reversible two electron transfer processes have been observed /7d/. The anodic peak potentials *vs.* $Cp_2Fe^{0/+}$ were recorded at -785 mV and -1130 mV, respectively. The difference correlates well with the electronic structure of the bridging unit. Ligand $7^{4-}$ with an electron donating alkyl bridge creates a more electron rich environment compared to $6^{4-}$ with the electron withdrawing diamide bridge. Thus, complexes with $7^{4-}$ show a more negative reduction potential. The substitution of a diamide bridge for an alkyl bridge in the nickelate complex anions leads to a potential shift of approximately -350 mV. The anodic peak potential of the corresponding cobalt complex $(NEt_4)_2[Co_2(\mathbf{6})_2]$ **15** was found at -1160 mV /7d/. The change of the metal center in isostructural complexes from nickel to cobalt leads again to a potential shift of -375 mV. Thus, the change from the diamide linked ligand $\mathbf{6}^{4-}$ to the alkyl linked ligand $\mathbf{7}^{4-}$ effects the $Ni^{II}/Ni^{III}$ potential in a comparable way to the substitution of the metal center from nickel to cobalt in complexes of $\mathbf{6}^{4-}$. These results corroborate the proposal /9/ that the SOMO in bis(enedithiolate) complexes carries both metal and ligand character. The $Ni^{III}/Ni^{IV}$ redox couple, reported by Sellmann *et al.* /10/ for the corresponding complex with 3,5-Di(*tert*-butyl)benzene-1,2-dithiolate was not observed.

The fact that each species exhibits only one redox wave leads to the assumption that no strong intramolecular interaction of the nickel centers exist in the dinuclear complexes. Consequently, no mixed valent species could be observed. However, a thorough analysis of the cyclic voltammograms discloses slight differences between the couple **13a/b** with the longer diamide bridge and the couple **14a/14b** with the shorter ethylene bridge. The difference of the peak potentials ΔE is close to ideal reversibility for **13a/13b** (70 mV). The nickel centers behave like independent mononuclear complexes. In contrast, ΔE for **14a/14b** amounts to 162 mV, which might indicate a slight nickel-nickel interaction in the anions $[Ni_2(\mathbf{7})_2]^{2-/4-}$ /7d/.

In contrast to the nickel compounds, complexes of iron(III) with bis(benzene-1,2-dithiolato) ligands form different structural motifs, which was shown by Sellmann with the isolation of $(Ph_4As)_2[Fe_2(\mathbf{10})_2]$ **16** (Figure 5) /11/. The tendency of iron(III) bis(1,2-dithiolato) complexes to dimerize via [Fe-S-Fe] bridges led to a more compact complex anion, in which two cofacial $Fe(S_2C_6H_3R)_2$ complex units are linked by a $\mu^3$-interaction of two sulfur atoms and two amide bridges. The possibility to incorporate substrates between the iron centers is impressively illustrated.

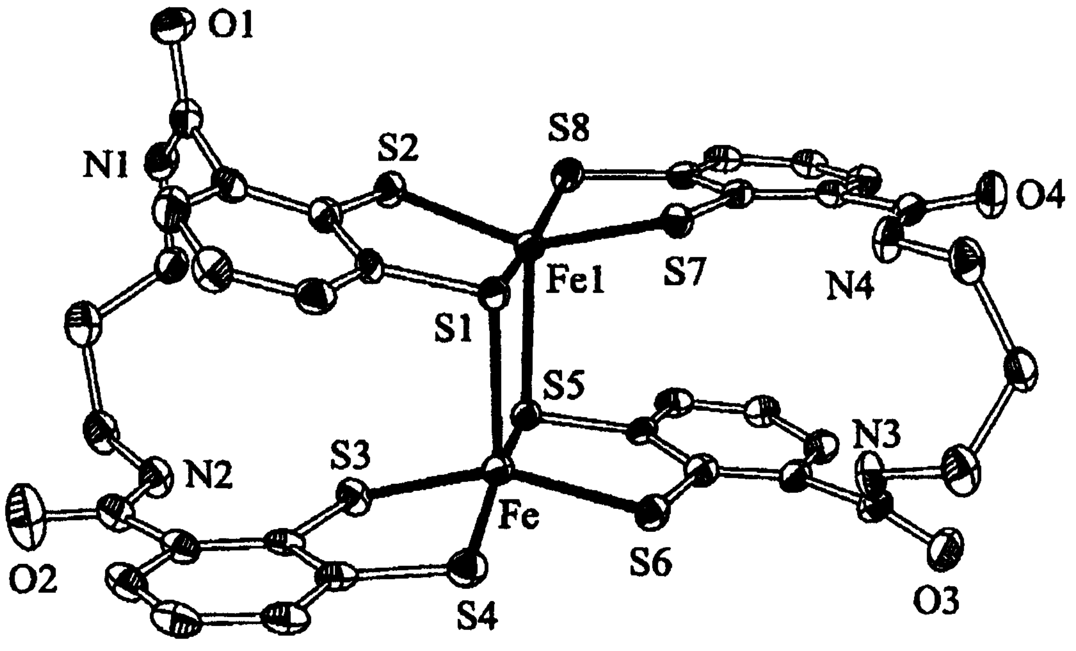

**Fig. 5:** Molecular structure of the complex anion in $(Ph_4As)_2[Fe_2(\mathbf{10})_2]$ **16**.

### TRIS(BENZENE-1,2-DITHIOLATO) COMPLEXES

Transition metal ions, which are known to form tris(benzene-1,2-dithiolato) complexes, are suitable candidates for the preparation of dinuclear triple-stranded derivatives. With respect to such tris complexes a particularly rich coordination chemistry has been described for molybdenum and tungsten /12/. In order to assess the tendency of the complex systems to form helical structures we completed the structural characterization of the series $[M(C_6H_4S_2\text{-}1,2)_3]^{n-}$ (n = 0, 1, 2) for molybdenum and tungsten by the detemination of the molecular structures of $[W^{VI}(C_6H_4S_2\text{-}1,2)_3]$ **17** /13/ and $(Ph_3PNPPh_3)_2[Mo^{IV}(C_6H_4S_2\text{-}1,2)_3]$ **18** /14/. The tungsten(VI) ion in **17** (Figure 6) is coordinated by six sulfur atoms of three benzene-1,2-dithiolato ligands in an almost perfect trigonal-prismatic fashion with a crystallographically imposed twist angle of 0°. The molecular geometry of complex **17** is remarkably similar to its lower homologue $[Mo^{VI}(C_6H_4S_2\text{-}1,2)_3]$ /12a/. The one electron reduction of trigonal prismatic **17** results in pseudooctahedral $(Ph_4As)[W^{V}(C_6H_4S_2\text{-}1,2)_3]$ /12c/ with an average twist angle of 33°. The pseudooctahedral coordination was also observed for the analogous $Mo^{V}$ complex /12b/. Here it was explained with the occupation of the $\pi$-antibonding $4a_1$' molecular orbital causing destabilization of both S···S interactions and M-S bonds and leading to an octahedral distortion /12b/ Following this argument for the tungsten complexes, the dianionic $[W^{IV}(C_6H_4S_2\text{-}1,2)_3]^{2-}$ should adopt a symmetry even closer to octahedral than the $W^{V}$ complex due to the fully occupied $4a_1$' orbital. Surprisingly, this assumption was disproved by the close trigonal-prismatic geometry found for $[W^{IV}(C_6H_4S_2\text{-}1,2)_3]^{2-}$ /12d/ with an average twist angle of only 3.5°. To complete the confusion the molecular structure of $[Mo^{IV}(C_6H_4S_2\text{-}1,2)_3]^{2-}$ in **18** displays remarkable similarity to the $Mo^{V}$ system, however with an average twist angle of 24.9° somewhat shifted towards trigonal-prismatic coordination. From the variation of the twist angles $\Phi$ in the series $[M(C_6H_4S_2\text{-}1,2)_3]^{n-}$ (M = Mo, W; n = 0, 1, 2) it appears that the d-electron configuration is not the only governing principle for the formation of a

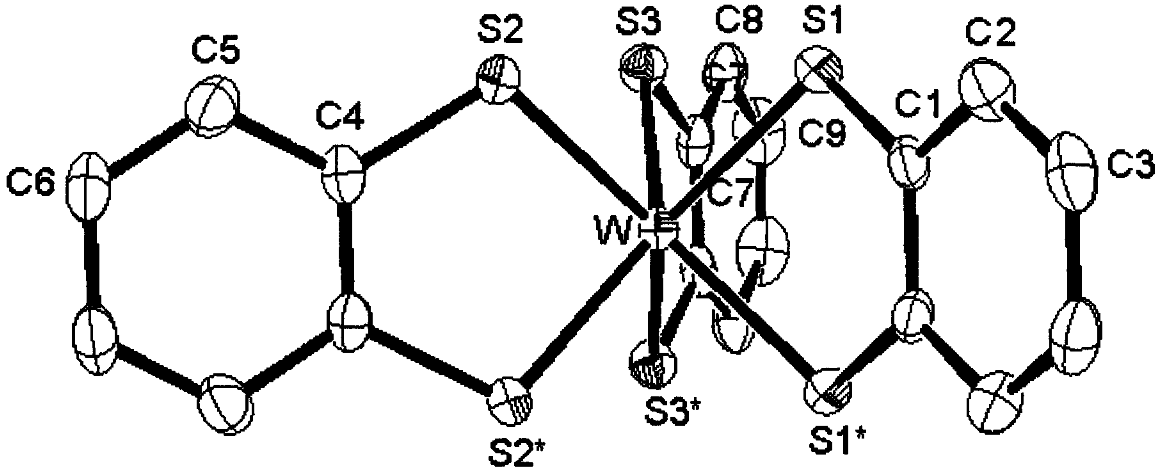

**Fig. 6:** Molecular structure of complex $[W^{VI}(C_6H_4S_2\text{-}1,2)_3]$ **17**.

certain coordination geometry. However, metal atoms with an odd valence electron number seem to enforce a more octahedral coordination, whereas the $d^0$-ions prefer a trigonal-prismatic structure. As a conclusion from this study triple-stranded tris(benzene-1,2-dithiolato) complexes with $\mathbf{6}^{4-}$, $\mathbf{7}^{4-}$ or $\mathbf{10}^{4-}$ can be expected to adopt a non helical geometry with $Mo^{VI}/W^{VI}$, whereas helical coordination compounds should be formed with $Mo^{V}/W^{V}$.

The synthetic procedures for the preparation of the neutral metal(VI) complexes (e.g. reaction of $WMe_6$ with benzene-1,2-dithiol in diethylether) cannot be transferred to the bis(benzene-1,2-dithiol) ligands because of their poor solubility. However, synthesis of the dinuclear complex anions $[Mo_2(\mathbf{6})_3]^{4-}$ can be achieved by straightforward metathesis reaction of two equivalents of $MoCl_4$(dme) with three equivalents of $Li_4$-**6** applying thermodynamic equilibration conditions. The salt $Li_4[Mo_2(\mathbf{6})_3]$ has been characterized spectroscopically and the dinuclear nature of the bulk product has been demonstrated by ESI mass spectroscopy /15/. However, due to the high tendency of salts containing the anion $[Mo_2(\mathbf{6})_3]^{n-}$ to form intensely colored, blue or purple glasses, we have not been successful in their structural characterization so far.

### SECOND GENERATION LIGANDS

Due to the high thermodynamic stability of mononuclear bis(benzene-1,2-dithiolato) complexes of iron we encountered problems during our attempts to coordinate a tetradentate bis(benzene-1,2-dithiolato) ligand to a $Fe(\mu\text{-}S)_2Fe$ moiety. Even cautious ligand transfer reactions with $[Fe_2S_2Cl_4]^{2-}$ or with $[Fe_2S_2\{C_6H_4(CH_2S)_2\}_2]^{2-}$/16/ led to species, which no longer contained sulfide ions. In our approach to tackle that problem we designed comparable bulky bis(benzene-1,2-dithiolato) ligands with a high degree of preorganization based on the dimercaptoterephthalic acid. The terephthalic acid derivative **19** (Figure 7) can be prepared in a procedure similar to that outlined in Scheme 1 by double *o*-lithiation of dilithium-benzene-1,2-dithiolate, treatment with $CO_2$ and subsequent trapping with benzylbromide. The use of **19** instead of **2** allows the double-bridging of two benzenedithiol units. The formation of tetraamide macrocycles like **20** applying template techniques turned out to be the crucial step in the ligand synthesis. The bis(benzene-1,2-dithiol) **20** (Figure 7) is furnished with two rigid 1,1'-biphenyl-4,4'-bis(methyl-N-carboxamide) bridges, which keeps the two benzene-1,2-dithiolato donors at an appropriate distance to incorporate the $Fe(\mu\text{-}S)_2Fe$ unit. Investigations regarding the coordination chemistry of **20** are currently underway.

### ACKNOWLEDGEMENT

We thank the Deutsche Forschungsgemeinschaft and the Fonds der Chemischen Industrie for financial support.

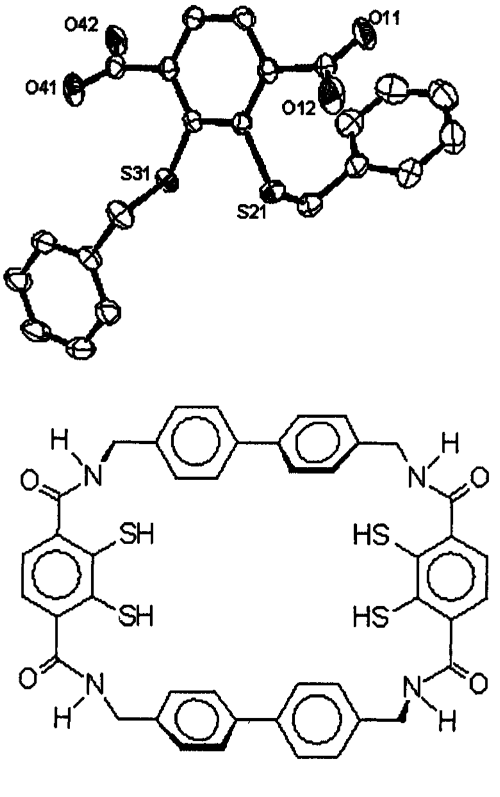

20

**Fig. 7:** Molecular structure of **19** (top) and macrocyclic ligand **20** (bottom).

### REFERENCES

1. a) R. P. Burns, C. A. McAuliffe, *Adv. Inorg. Chem. Radiochem.*, **22**, 303 (1979); b) C. Mahadevan, *J. Crystallogr. Spectrosc. Res.*, **16**, 347 (1985); c) U. Mueller-Westerhoff, B. Vance *Comprehensive Coordination Chemistry vol. 2*, Pergamon **1987**, 516; d) P. Cassoux, *Coord. Chem Rev.*, **185-186**, 213 (1999).
2. a) E. I. Stiefel, H. B. Gray, *J. Am. Chem. Soc.*, **87**, 4012 (1965); b) M. J. Baker-Hawkes, E. Billig, H. B. Gray, *J. Am. Chem. Soc.*, **88**, 4870 (1966); c) M. J.Bennett, M. Cowie, J. L. Martin, J. Takats, *J. Am. Chem. Soc.*, **95**, 7504 (1973); d) G. Henkel, B. Krebs, *Angew. Chem.*, **97**, 113 (1985); *Angew. Chem. Int. Ed.*, **24**, 117 (1985); e) S. Boyde, S. R. Ellis, C. D. Garner, W. Clegg, *J. Chem. Soc., Chem. Commun.*, **1986**, 1541; f) T. R. Halbert, E. I. Stiefel, *Inorg. Chem.*, **28**, 2501 (1989); g) J. P. Donahue, C. R. Goldsmith, U. Nadiminti, R. H. Holm, *J. Am. Chem. Soc.*, **120**, 12869 (1998); h) T. Matthew Cocker, R. E. Bachman, *J. Chem. Soc., Chem. Commun.*, **1999**, 875; i) B. S. Lim, M. W. Willer, M. Miao, R. H. Holm, *J. Am. Chem. Soc.*, **123**, 8343 (2001); j) A. Cervilla, F. Perez-Pla, E. Llopis, *J. Chem. Soc., Chem. Commun.*, **2001**, 2332; k) T. B. Rauchfuss, S. M. Contakes, S. C. N. Hsu, M. A. Reynolds, S. R. Wilson, *J. Am. Chem. Soc.*, **123**, 6933 (2001); l) X.-B. Wang F. E. Inscore, X. Yang, J.

J. A. Coonex, J. H. Enemark, L.-S. Wang, *J. Am. Chem. Soc.*, **124**, 10182 (2002).

3. a) R. Cammack, *Adv. Inorg. Chem.*, **38**, 281 (1992); b) W. R. Rypniewski, D. R. Bre iter, M. M. Benning, G. Wesenberg, B.-H. Oh, J. L. Markley, I. Rayment, H. M. Holden, *Biochemistry*, **30**, 4126 (1991).
4. S. Iwata, M. Saynovits, T. A. Link, H. Michel, *Structure*, **4**, 567 (1996).
5. a) R. C. Scarrow, D. L. White, K. N. Raymond, *J. Am. Chem. Soc.*, **107**, 6540 (1985); b) T. Beissel, R. E. Powers, K. N. Raymond, *Angew. Chem.*, **108**, 1166 (1996); *Angew. Chem. Int. Ed.*, **35**, 1084 (1996); c) D. L. Caulder, K. N. Raymond, *Angew. Chem.*, **109**, 1508 (1997); *Angew. Chem. Int. Ed*, **36**, 1440 (1997); d) B. Kersting, M. Meyer, R. E. Powers, K. N. Raymond, *J. Am. Chem. Soc.*, **118**, 7221 (1996); e) M. Albrecht, S. Kotila, *Angew. Chem.*, **107**, 2285 (1995); *Angew. Chem. Int. Ed.*, **34**, 2134 (1995); f) M. Albrecht, *Synthesis*, **1995**, 230; g) E. J. Enemark, T. D. P. Stack, *Angew. Chem.*, **107**, 1082 (1995); *Angew. Chem. Int. Ed..*, **34**, 996 (1995); h) M. A. Masood, E. J. Enemark, T. D. P. Stack, *Angew. Chem.*, **110**, 973 (1998); *Angew. Chem. Int. Ed..*, **37**, 928 (1998); i) M. Albrecht, *Chem. Soc. Rev.*, **27**, 281 (1998).
6. C. Piguet, G. Bernadinelli, G. Hopfgartner, *Chem. Rev.*, **97**, 2005 (1997).
7. a) F. E. Hahn, W. W. Seidel, *Angew. Chem.*, **107**, 2938 (1995); *Angew. Chem. Int. Ed.*, **34**, 2938 (1995); b) W. W. Seidel, F. E. Hahn, T. Lügger, *Inorg. Chem.*, **37**, 6587 (1998); c) W. W. Seidel, F. E. Hahn, *J. Chem. Soc. Dalton Trans.*, **1999**, 2237; d) H. V. Huynh, C. Schulze-Isfort, W. W. Seidel, T. Lügger, R. Fröhlich, O. Kataeva, F. E. Hahn, *Chem. Eur. J.*, **8**, 1327 (2002); e) H. V. Huynh, W. W. Seidel, T. Lügger, R. Fröhlich, B. Wibbeling, and F. E. Hahn, *Z. Naturforsch.*, **57b**, 1401 (2002).
8. H. Köpf, K. Lange, J. Pickhardt, *J. Organomet. Chem.*, **420**, 345 (1991).
9. B. S. Lim, D. V. Formitchev, R. H. Holm, *Inorg. Chem.*, **40**, 4257 (2001).
10. D. Sellmann, H. Binder, D. Haussinger, F. W. Heinemann, J. Sutter, *Inorg. Chim. Acta*, **300**, 829 (2000).
11. D. Sellmann, K. P. Peters, R. M. Molina, F. W. Heinemann, *Eur. J. Inorg. Chem.*, **2003**, 903.
12. a) M. Cowie, M. J. Bennett, *Inorg. Chem.*, **15**, 1584 (1976); b) A. Cervilla, E. Llopis, D. Marco, F. Perez, *Inorg. Chem.*, **40**, 6525 (2001); c) T. E. Burrow, R. H. Morris, A. Hills, D. L. Hughes, R. L. Richards, *Acta Crystallogr.*, **C49**, 1591 (1993); F. Knoch, D. Sellmann, W. Kern, *Z. Kristallogr.*, **202**, 326 (1992); d) C. Lorber, J. P. Donahue, C. A. Goddard, E. Nordlander, R. H. Holm, *J. Am. Chem. Soc.*, **120**, 8102 (1998).
13. H. V. Huynh, T. Lügger, F. E. Hahn, *Eur. J. Inorg. Chem.*, **2002**, 3007.
14. C. Schulze-Isfort, T. Lügger, F. E. Hahn, unpublished results
15. H. V. Huynh, F. E. Hahn, unpublished results
16. J. J. Mayerle, S. E. Denmark, B. V. DePamphilis, J. A. Ibers, R. H. Holm, *J. Am. Chem. Soc.*, **97**, 1032 (1975).

